# Phytochemical Characterization and Antioxidant, Antimicrobial, and Poultry Meat Preservation Potential of *Ziziphus mauritiana*

**DOI:** 10.3390/foods15071193

**Published:** 2026-04-01

**Authors:** Mohamed Gamal Shehata, Hassan Mohamed Al Marzooqi, Hanan Sobhy Afifi, Saad H. D. Masry

**Affiliations:** 1 Food Research Section, Applied Research and Capacity Building Division, Abu Dhabi Agriculture and Food Safety Authority (ADAFSA), Abu Dhabi 52150, United Arab Emirates; hassan.marzouqi@adafsa.gov.ae (H.M.A.M.); hanan.afifi@adafsa.gov.ae (H.S.A.); 2 Food Technology Department, Arid Lands Cultivation Research Institute (ALCRI), City of Scientific Research and Technological Applications (SRTACity), New Borg El-Arab 21934, Egypt; 3 Agriculture Research Section, Applied Research and Capacity Building Division, Abu Dhabi Agriculture and Food Safety Authority (ADAFSA), Al Ain 52150, United Arab Emirates; saad.masry@adafsa.gov.ae; 4 Department of Plant Protection and Molecular Diagnosis, Arid Lands Cultivation Research Institute, City of Scientific Research and Technological Applications (SRTACity), Alexandria 21934, Egypt

**Keywords:** *Ziziphus mauritiana*, phenolic compounds, antioxidant activity, antimicrobial activity, HPLC, chicken meat, natural preservatives

## Abstract

Consumer preference for clean-label products is driving interest in natural antioxidants and antimicrobials that can replace synthetic preservatives. *Ziziphus mauritiana* (sidr), a resilient desert tree native to the arid Gulf region, has being tested as a multifunctional bio-preservative. This study evaluated the extraction yield, total phenolic content (TPC), total flavonoid content (TFC), antioxidant and antimicrobial activities, and poultry meat-preserving potential of *Z. mauritiana.* Methanol and ethanol produced the highest extract recoveries, with bark exhibiting the maximum extraction yield of up to 10.7 mg/100 g. Fruits demonstrated the highest total phenolic content (TPC) of around 175 mg gallic acid equivalents per gram (GAE/g) and total flavonoid content (TFC) of around 7.4 mg catechin equivalents per gram (CE/g), followed by leaves and bark. The antioxidant activity was significantly correlated with the concentration of phenolic compounds in the fruit extracts, which exhibited DPPH inhibition exceeding 60% in the majority of instances. The RP-HPLC investigation revealed a diverse polyphenolic profile, predominantly featuring gallic acid (up to 8.77 mg/g in leaves), catechin (6.30 mg/g in fruits), catechol, and caffeic acid. Leaf extracts showed 24 mm inhibitory zones against *E. coli* and *Y. enterocolitica*, while bark and fruit were not very effective. Adding ethanolic leaf extract (0–1%) to chicken breast meat reduced microbial degradation during chilled storage at 4 °C. At day 15, total aerobic counts reached only 5.34 log CFU/g with 1% extract compared with 8.53 log CFU/g in the control. Similar suppression was found for yeasts and molds, while challenge tests showed >3-log reductions in *C. jejuni* and *Salmonella senftenberg*. Sensory evaluation confirmed no detrimental effects on color, odor, flavor, or texture. Overall, *Z. mauritiana* was a valuable, renewable source of phenolic antioxidants and antimicrobial agents and showed strong promise as a natural preservative capable of improving the safety and shelf life of poultry meat in clean-label applications.

## 1. Introduction

Consumer preference for minimally processed foods has accelerated the search for preservatives sourced from plants. Although widely used synthetic agents such as BHT and nitrites are effective, continuing toxicological concerns and evolving regulatory expectations have strengthened the case for less hazardous alternatives obtainable from edible or medicinal species [1]. Plant-derived antioxidants can limit lipid oxidation, helping to preserve product quality and sensory characteristics. Importantly, their relevance extends beyond the food matrix, as antioxidant activity is also associated with reduced oxidative stress and may help lower the burden of chronic conditions linked to free-radical processes [2].

Polyphenols, particularly flavonoids and phenolic acids, are key bioactive constituents responsible for the antimicrobial and antioxidant properties of many plant extracts. Their ability to neutralize free radicals and inhibit microbial growth is especially relevant in meat systems, where lipid oxidation and microbial spoilage occur simultaneously and significantly reduce product quality and safety [3]. Therefore, identifying phenolic-rich plant sources with dual antioxidant–antimicrobial functionality is of particular importance for the development of clean-label preservation strategies [4].

Conventional preservation methods for chicken filets include refrigeration, modified atmosphere packaging (MAP), vacuum packaging, synthetic antioxidants and antimicrobials, irradiation, and high-pressure processing [3]. Although these approaches can effectively delay microbial growth and oxidative deterioration, their overall effectiveness during extended storage may be limited by residual microbial activity and progressive lipid oxidation. Chemical preservatives such as nitrites and synthetic antioxidants (e.g., BHT, BHA) can enhance stability; however, concerns regarding potential toxicological effects, regulatory constraints, and growing clean-label consumer expectations have encouraged the search for safer alternatives. Additionally, advanced technologies such as irradiation and high-pressure processing, while effective, may increase production costs and require specialized infrastructure. Consequently, increasing demand for minimally processed and clean-label foods has stimulated interest in natural plant-derived bioactive compounds as sustainable preservation strategies [4].

The United Arab Emirates, and Abu Dhabi in particular, is characterized by an arid desert climate marked by extreme temperatures, intense solar irradiation, and limited water availability. Such conditions favor plant species that tolerate multiple abiotic stresses, often through enhanced production of secondary metabolites—including phenolics, tannins, and flavonoids—that contribute to ecological resilience and are also of interest for their biological activity in humans [5]. The arid-region tree *Ziziphus mauritiana* Lam., typically referred to as sidr or Indian jujube, is a notable instance. Widely utilized in traditional medicine for its fruits, leaves, and bark, *Z. mauritiana* is recognized for its antioxidant, hepatoprotective, anti-inflammatory, and antimicrobial properties, which can be largely attributed to its phenolic profile [6]. Beyond pharmacological applications, limited studies have explored the food preservation potential of *Ziziphus* species. Extracts from *Ziziphus jujuba* and *Ziziphus mauritiana* have demonstrated antimicrobial activity against foodborne pathogens and antioxidant activity in model food systems, suggesting potential applicability in meat preservation. However, systematic evaluations combining phytochemical profiling with practical application in real poultry matrices remain scarce [7,8].

Previous research has suggested sizeable variations in the phytochemical composition of *Ziziphus* spp. as a function of genotype, geographical origin, plant part, and extraction conditions. In particular, leaves and fruits have repeatedly been shown to contain high levels of flavonoids, phenolic acids and saponins, which not only contribute to antioxidant capacity but may additionally underlie the reported antibacterial, antifungal and antiparasitic results [9]. However, the majority of available work has been targeted on medicinal or pharmacological aspects, frequently using crude extracts without detailed profiling of individual phenolic compounds. Comprehensive studies that integrate solvent-dependent extraction behavior, quantitative TPC and TFC, detailed chromatographic phenolic profiling, and functional antioxidant and antimicrobial assays remain comparatively scarce for *Z. mauritiana*, particularly within the context of arid Gulf environments [10].

Several plant extracts have been successfully applied in poultry meat systems. Rosemary and green tea extracts have reduced lipid oxidation and total viable counts in refrigerated chicken filets, while pomegranate peel and grape seed extracts have demonstrated both antimicrobial effects and sensory stability during storage [11,12]. Essential oils from oregano and thyme have also shown inhibitory effects against *Salmonella* and *Campylobacter* in poultry matrices [13]. These studies have provided strong evidence that phenolic-rich plant extracts can function as multifunctional bio-preservatives in real meat systems.

Chicken meat is particularly prone to quality deterioration due to its relatively high content of unsaturated fatty acids compared with other meat types, which increases its susceptibility to lipid oxidation, as well as its vulnerability to contamination by pathogens such as *Campylobacter jejuni* and *Salmonella* spp. Conventional preservation techniques depend heavily on refrigeration combined with chemical additives; however, there is growing pressure to replace or reduce synthetic with natural alternatives [14]. Plant extracts combining antioxidant and antimicrobial activities are well suited to this purpose, provided they do not adversely affect sensory attributes such as color flavor and texture [15].

Despite the reported bioactivity of *Ziziphus* spp., comprehensive studies integrating solvent-dependent phytochemical profiling, genotype comparison, pathogen challenge validation, and application in real poultry matrices remain limited. Accordingly, the present study aimed to: (i) quantify the extraction yield and the total phenolic and flavonoid contents of *Ziziphus mauritiana* leaves, bark and fruits extracted with water, ethanol and methanol; (ii) characterize their antioxidant potential and relate it to phenolic profiles determined by RP-HPLC; (iii) compare the antimicrobial activity of the extracts against important food-borne pathogens; and (iv) investigate the effectiveness of *Ziziphus mauritiana* Lam. leaf extract as a natural bio-preservative in chilled chicken meat, including its effect on microbial load, pathogenic challenge tests and sensory quality.

## 2. Materials and Methods

### 2.1. Plant Material and Sampling Locations

Mature *Ziziphus mauritiana* trees were sampled from four locations within the Abu Dhabi Emirate: Baniyas Research Station, Kuwaitat Research Station, Salamat Research Station and a private farm at Abu Koriya, Al-Ain. These locations represent distinct micro-environments within the Emirate and encompass different *Ziziphus mauritiana* Lam. genotypes: a local cultivar at Baniyas and Kuwaitat, a Pakistani origin at Salamat, and a Thai variety at Abu Koriya, Al-Ain Farm. All chemicals and reagents used in this study were purchased from Sigma-Aldrich (St. Louis, MO, USA).

From each location, leaves, bark, and fruits were collected manually from healthy trees showing no visible signs of disease, pathogen infection, nutrient deficiency, or abiotic stress at the time of sampling. For each organ, composite samples were obtained by pooling material from several trees to minimize intra-tree variability. Plant material was transported to the laboratory in clean polyethylene bags and processed within 24 h. All sites were visited during the fruiting season; however, fruit samples were not available at the Baniyas location at the time of collection.

Upon arrival, leaves and bark were washed with distilled water to remove dust and surface contaminants. Samples were air-dried under shaded laboratory conditions at 25 ± 2 °C with continuous air circulation for 6 days until constant weight was achieved. Moisture stabilization was confirmed by successive weight measurements at 24 h intervals. Dried samples were ground using a laboratory stainless-steel grinder (Model HR2726, Philips, Shanghai, China) and sieved through a 60-mesh sieve (250 µm) to obtain a uniform particle size. The final granulometry was approximately ≤250 µm. The powdered samples were stored in airtight, opaque containers at room temperature (25 ± 2 °C) until extraction. The storage procedure followed standard practices for phenolic stability as previously described [12].

### 2.2. Extracts Preparation

Extract preparation was performed according to Hegazy and Ibrahium [16]. Twenty grams of the dried, finely ground powder of each plant part (leaves, bark, or fruits) were extracted with 200 mL of distilled water, ethanol, or methanol (1:10 *w*/*v*) at room temperature (25 ± 2 °C) using the maceration method for 6 h. Extracts were filtered through a Whatman No. 2 filter paper for the removal of particles. The residue was re-extracted twice under the same condition to ensure complete extraction. Yield of the components in different solvents was estimated by evaporating the organic solvents under vacuum using a rotary evaporator (Buchi R-210, Büchi Labortechnik AG, Flawil, Switzerland), followed by lyophilization. Extraction yield (%) was calculated according to the following equation: yield (%) = (weight of dried extract (g)/weight of initial dry plant material (g)) × 100

### 2.3. Determination of Total Phenolic and Total Flavonoid Contents

Total phenols content (TPC) were determined according to Singleton and Rossi [17]. Briefly, 50 μL of extracts were mixed with 3 mL of deionized water and 250 μL of Folin–Ciocalteu reagent (1 N). After 8 min of equilibrium, 750 μL of 20% Na_2_CO_3_ and 950 μL of H_2_O were added to the extracts; after incubation for 30 min at room temperature (25 ± 2 °C), the absorbance was read at 765 nm with a UV-Vis spectrophotometer (UV-2505, Labomed, Inc., Los Angeles, CA, USA). The concentration of total phenols content compound was calculated using a standard curve of aqueous solutions of gallic acid and expressed as mg gallic acid equivalent/g dry weight of extract (mg GAE/g DW). The total flavonoid content (TFC) was determined according to methods described by Gonzalez-Aguilar et al. [18]. In total, 1 mL from each extracted sample was mixed and equilibrated with 4 mL of deionized water and 300 μL 5% NaNO_2_ for 5 min. After equilibrium, 300 μL of 10% AlCl_3_ (methanolic solution) were added; the mixture was allowed to sit for 1 min and then 2 mL of 1 M NaOH were added. The last volume was completed to 10 mL with H_2_O, stirred, and readings were taken. The mixture absorbance was determined at 415 nm using a UV-Vis spectrophotometer. The concentration of total flavonoids of fruits was calculated using a standard curve of catechol and expressed as mg catechol equivalent/100 g dry weight of the extract (mg CE/g DW).

### 2.4. Antioxidant Activity: DPPH Radical-Scavenging Assay

The DPPH (2,2-diphenyl-1-picrylhydrazyl) assay was used to measure free radical-scavenging activity, following Brand-Williams et al. [19] with minor adjustments. Each extract solution (1 mL at different concentrations) was combined with 1 mL of methanolic DPPH solution (0.078 mM). The mixture was shaken thoroughly and incubated (Memmert IN55, Memmert GmbH, Schwabach, Germany) at room temperature (25 ± 2 °C) in the dark for 30 min, after which the absorbance was measured at 517 nm. Methanolic DPPH solution without extract served as control.

The percentage inhibition of DPPH radicals was calculated as:

The inhibition of DPPH radical (%) = [(Abs_control_ − Abs_sample_)]/(Abs_control_)] × 100.

where A_control_ is the absorbance of the control solution and A_sample_ is the absorbance of the test extract.

### 2.5. HPLC Analysis of Phenolic Compounds

The phenolic profiles of the selected ethanolic extracts were determined by RP-HPLC following the method of Tomaino et al. [20], with specific chromatographic conditions specified in the technical report. A Waters system (Waters, Milford, MA, USA) equipped with a UV/VIS detector set at 280 nm and a Shimadzu Pathfinder^®^ AS silica 100 RP column (150 × 4.6 mm, 5 µm, Shimadzu, Kyoto, Japan) were used. The mobile phase comprised 0.01% acetic acid in water (solvent A) and a mixture of methanol, acetonitrile, and acetic acid at a ratio of 95:5:1 *v*/*v*/*v* (solvent B). A gradient elution was performed from 5% to 100% mobile phase B over 50 min. The mobile phase A consisted of water containing 0.1% formic acid, and the mobile phase B consisted of acetonitrile containing 0.1% formic acid. The flow rate was maintained at 1.0 mL/min, the column temperature was set at 30 °C, and the injection volume was 20 µL. After each run, the column was returned to the initial conditions and allowed to re-equilibrate prior to the next injection. Quantification was performed using external calibration with individual phenolic reference compounds, including gallic acid, catechol, p-hydroxybenzoic acid, catechin, and caffeic acid. Data collection and subsequent processing were conducted using Waters Baseline 815 software. Analyte identification relied on agreement between the retention times and spectral characteristics of sample peaks and those obtained from the corresponding standards. For each compound, calibration curves were prepared independently and used for concentration determination.

### 2.6. In Vitro Antimicrobial Activity

The antimicrobial activities of the *Ziziphus mauritiana* Lam. extracts were evaluated using the agar-well diffusion method in line with Shehata et al. [21]. Extract solutions (100 mg/mL, dry extract basis) were prepared in the appropriate solvent, and 100 µL was dispensed into each 6 mm agar well. Six pathogenic strains were tested: *Escherichia coli* BA 12296, *Salmonella senftenberg* ATCC 8400, *Campylobacter jejuni* ATCC 700819, *Bacillus cereus* ATCC 49064, *Yersinia enterocolitica* ATCC 23715, and *Staphylococcus aureus* NCTC 10788. All bacterial strains were obtained from the Microbiology Laboratory, Faculty of Agriculture, Ain Shams University, Cairo, Egypt.

Bacterial cultures were grown to ~10^8^ CFU/mL, and 100 µL of each inoculum was mixed with the respective molten selective or general medium and poured into Petri dishes. After solidification, wells were punched into the agar and filled with 100 µL of extract solution at standardized concentration. The plates were incubated (Memmert IN55, Memmert GmbH, Germany) at 37 °C for 24 h, and the diameter of the inhibition zones (mm) around each well was measured. Antimicrobial activity was evaluated using ethanolic extracts. After solvent was removed under reduced pressure, the dried extracts were reconstituted in sterile distilled water to the desired concentrations prior to testing. A solvent control (sterile distilled water) was included to verify that inhibition zones were attributable to the extract rather than residual solvent effects. The experiments were performed in triplicate.

### 2.7. Application in Chilled Chicken Meat

Fresh boneless, skinless chicken breast filets were purchased from a certified local poultry retailer in Abu Dhabi, UAE, within 24 h post-slaughter. The meat was supplied in its original food-grade polyethylene packaging under refrigerated conditions (4 ± 1 °C). Samples were transported to the laboratory in insulated containers with ice packs and processed within 2 h of purchase. Prior to treatment, the meat was stored at 4 °C and handled under aseptic laboratory conditions.

#### 2.7.1. Microbiological Profile Analysis of Raw Chicken Meat Products

Ten grams of raw chicken meat products were examined at different intervals (0, 3, 6, 9, 12, and 15 days) during storage at 4 °C. The samples were first diluted in 90 mL of sterile saline solution (0.9% *w*/*v*), following the procedure reported by Polychroniadou et al. [22], and homogenized for 2 min using a laboratory stomacher under aseptic conditions. Plate Count Agar (PCA; Himedia, Mumbai, India) was used for the enumeration of aerobic mesophilic bacteria. The inoculated plates were incubated (Memmert IN55, Memmert GmbH, Germany) at 30 °C for 48 h before counting. *Salmonella* was determined using SS agar (Himedia, India), whereas coliform bacteria were determined on Violet Red Bile Agar (VRBA; Himedia, India). In both cases, incubation was carried out at 37 °C for 48 h. Yeasts and molds were counted on Potato Dextrose Agar (PDA; Himedia, India) after incubation at 30 °C for a period ranging from 48 to 72 h. All microbiological assessments were carried out using the pour plate technique as outlined by Abd El-Aziz et al. [23]. The microbial counts were reported as log10 colony-forming units per gram (log10 CFU/g) of the raw chicken meat products. The experiment was conducted using three independent batches of chicken meat prepared on separate days. For each treatment group, samples were analyzed in triplicate at each storage interval. All microbiological analyses were performed in duplicate plates per dilution, and the entire experiment was repeated twice to ensure reproducibility.

#### 2.7.2. In Vitro Antimicrobial Assay and Challenge Study in Chicken Meat

Approximately 100 g portions of fresh chicken breast meat were aseptically packaged in sterile polyethylene bags (Hotpack, Abu Dhabi, United Arab Emirates). To evaluate the in situ antimicrobial efficacy of *Ziziphus mauritiana* leaf extract, a controlled challenge study was conducted using *Campylobacter jejuni* ATCC 700819 and *Salmonella senftenberg* ATCC 8400.

For each pathogen, meat samples were randomly assigned to three treatments: (i) uninoculated control, (ii) pathogen-inoculated control (without extract), and (iii) pathogen-inoculated samples treated with 1% (*w*/*w*) *Z. mauritiana* ethanolic leaf extract.

Bacterial inocula were prepared in sterile saline to a defined concentration (CFU/mL), initially estimated by optical density, and subsequently confirmed by plate enumeration. An appropriate volume of each suspension was aseptically applied to the meat surface to obtain a target contamination level of approximately 7 log_10_ CFU/g. This inoculation level was selected in accordance with established microbial challenge-test protocols simulating high contamination scenarios in meat systems [24]. The final microbial concentration was calculated based on inoculum density (CFU/mL), inoculation volume (mL), and sample weight (g). Each pathogen was inoculated separately.

Immediately after inoculation, the treated samples received the designated concentration of *Z. mauritiana* extract and were manually homogenized to ensure uniform distribution. Samples were then stored at 4 °C for 15 days.

Microbiological analyses were performed on days 0, 3, 6, 9, 12, and 15 of storage. Viable counts of *C.jejuni* were enumerated on Mueller–Hinton Agar (Sigma-Aldrich, St. Louis, MO, USA) following incubation at 37 °C for 24 h under microaerophilic conditions. *Salmonella senftenberg* was enumerated using SS agar (Merck, Darmstadt, Germany) after incubation at 37 °C for 24 h. The results were expressed as log_10_ CFU/g of chicken meat. All analyses were performed in triplicate.

### 2.8. Sensory Evaluation

Fifteen participants (average age = 35 years old) were enrolled in a panel test on cooked meat chicken at the Food Research section, Applied Research and Capacity Building Division, ADAFSA, Abu Dhabi, UAE, which was conducted as previously described Tura et al. [25]. According to Damaziak et al. [26], sensory evaluation was performed on cooked chicken samples. The meat portions (approximately 20 g, 2 × 2 × 1 cm cubes) were cooked in a preheated electric oven at 180 °C until an internal temperature of 75 °C was reached. The samples were allowed to equilibrate to 40–45 °C before serving.

Each sample was presented on a white odorless disposable dish coded with random three-digit numbers. The evaluations were conducted under controlled laboratory conditions (22 ± 2 °C, neutral lighting).

Panelists were instructed to evaluate color, odor, taste, texture, appearance, and overall, acceptability using a 9-point hedonic scale, where 1 = dislike extremely, 2 = dislike greatly, 3 = dislike moderately, 4 = dislike slightly, 5 = neither dislike nor like, 6 = like slightly, 7 = like moderately, 8 = like greatly, and 9 = like extremely. Potable water was provided for palate cleansing between samples. Sensory evaluation was only performed at the initial stage (day 0) to determine the immediate impact of extract incorporation on product acceptability. All participants provided informed consent prior to participation. They were informed about the nature of the study, the type of samples evaluated, and their right to withdraw at any time without consequences.

### 2.9. Statistical Analysis

Data were analyzed using one-way analysis of variance (ANOVA) followed by Tukey’s post hoc test for multiple comparisons at a significance level of *p* < 0.05 [27]. All assays were performed in triplicate, and the results were expressed as mean ± standard deviation (SD). Statistical analyses were conducted using IBM SPSS Statistics for Windows (Version 23.0; IBM Corp., Armonk, NY, USA).

## 3. Results and Discussion

### 3.1. Extraction Yield: Influence of Plant Part, Solvent, and Genotype

Extraction yield is a primary determinant of the technological and economic feasibility of using plant extracts as food ingredients. In the present work, *Ziziphus mauritiana* Lam. bark consistently showed higher extraction yields than leaves and fruits across the different locations, irrespective of the solvent used, although the magnitude of difference varied (Table 1). At Baniyas, the yield from native bark varied from 9.72 mg/100 g (water) to 10.71 mg/100 g (methanol), while leaves produced 8.16–9.26 mg/100 g and fruits approximately 6.94–7.44 mg/100 g. Similar patterns were seen in Kuwaitat and Salamat for local and Pakistani genotypes, and in Abu Koriya for the Thai variety. Bark extracts gave substantially higher yields than fruit extracts. This disparity likely indicated the elevated concentrations of structural and storage phenolics in bark—substances such as tannins and lignin derivatives that exhibit good solubility in hydro-alcoholic solvents [4]. Similar observations have been reported for phenolic-rich woody tissues, where hydro-alcoholic solvents demonstrated superior extraction efficiency compared with aqueous systems due to improved solubilization of moderately polar compounds [4,8,10].

Fruits contain plenty of soluble phenolics too, but they are accompanied by sugars and organic acids. These additional compounds can co-precipitate with phenolics or get partially removed when extracts are concentrated and dried, lowering the final extract mass. The choice of solvent mattered. The higher extraction yields obtained with ethanol and methanol compared with water (*p* < 0.05) (Table 1) suggested that solvent polarity played a decisive role in recovering phenolic-rich fractions from *Z. mauritiana* tissues. The superior performance of ethanol was particularly relevant for potential food applications, as it combined extraction efficiency with food-grade suitability [28,29].

Genotypic differences were evident among the evaluated accessions. Local and Pakistani *Ziziphus mauritiana* Lam. samples generally exhibited higher extraction yields than the Thai variety, particularly in leaves and fruits (Table 1). Such variability may reflect genetic regulation of secondary metabolite biosynthesis as well as adaptive responses to arid environmental conditions. Previous studies on *Ziziphus* spp. have reported significant genotype-dependent variation in phenolic accumulation and extractable solids, influenced by both genetic background and ecological stress adaptation [30,31]. These findings supported the observed differences and suggested that locally adapted genotypes may represent promising sources of bioactive compounds for food applications.

### 3.2. Total Phenolic and Flavonoid Contents

TPC and TFC provide quantitative indices of the phenolic richness of plant extracts. In the current study, fruits of *Z. mauritiana* were clearly distinguished by their high phenolic load. For local fruits from Kuwaitat, the TPC values were 168.90 mg GAE/g DM in water, 171.50 mg GAE/g DM in ethanol, and 174.77 mg GAE/g DM in methanol extracts. The TFC values, on the other hand, were up to 7.41 mg catechol equivalents per gram of dry matter. Pakistani fruits from the same area also had higher TPC levels (up to 164.23 mg GAE/g DM) and TFC levels (above 5.8 mg/g) (Table 2). Fruits from Salamat and Abu Koriya had slightly lower but still significant values.

Leaves typically displayed intermediate TPC and TFC, though values remained high by comparison with many edible plants. For example, local leaves from Baniyas showed TPC between ~143 and 156 mg GAE/g DM and TFC between 3.5 and 4.7 mg/g across the different solvents, whereas Pakistani leaves from Salamat exhibited somewhat lower TPC (around 98–110 mg GAE/g DM) but comparable or higher TFC for some extracts (Table 2). Bark generally had lower TPC and TFC than leaves and fruits (e.g., 90–125 mg GAE/g DM and ~2–3.6 mg/g flavonoids in Salamat and Kuwaitat bark samples), though still at levels compatible with significant antioxidant activity.

*Ziziphus mauritiana* proved to be an excellent source of phenolic compounds, with fruits showing particularly high concentrations. Both TPC and TFC measurements revealed a clear pattern: fruits had the highest phenolic content, followed by leaves, and then bark. This distribution corresponded well with what each plant part did [30].

Fruits accumulate phenolics for several reasons; they protect against oxidative damage and microbial attack, and they contribute to characteristics like color and astringency that matter for the fruit’s role in seed dispersal. Leaves need phenolics mainly because they are exposed to intense light. The compounds can help protect leaf tissues from photo-oxidative damage that happens during photosynthesis [31]. Other studies on *Z. mauritiana* have reported comparable phenolic distributions across different plant organs. These findings reinforced the potential of fruits as the primary source of extractable phenolics for food and functional food applications [32,33]. Previous studies have shown that phenolic accumulation was organ-specific and functionally linked to plant defense strategies and physiological roles in fruits and leaves [1,8].

### 3.3. Antioxidant Activity

Antioxidant capacity was measured in *Z. mauritiana* extracts collected from several locations across Abu Dhabi Emirate, testing different plant parts and solvents (Table 3). The local accession had strong antioxidant activity. Ethanolic extracts from leaves reached 62.62 ± 1.01, while bark extracts reached 51.37 ± 0.76—both higher than methanol or water extracts. Ethanolic extracts exhibited significantly higher DPPH scavenging activity compared with aqueous extracts (*p* < 0.05), whereas methanolic extracts showed intermediate values (Table 3). These results indicated that solvent polarity significantly influenced antioxidant recovery. Phenolic compounds acted as hydrogen- or electron-donating agents that neutralize free radicals and stabilize reactive oxygen species, a mechanism widely reported for plant-derived antioxidants [34]. This suggested ethanol extracts antioxidant compounded more efficiently from these tissues [4]. At Kuwaitat Research Station, fruits gave the strongest results regardless of which solvent was used, with values between 63.85 ± 1.18 and 67.55 ± 0.91. Leaf and bark extracts were much weaker than fruit extracts, though ethanol still gave better yields than the other solvents. The Pakistani accession produced similar results. Among the plant materials examined, fruits consistently exhibited the highest antioxidant capacity across all comparisons. This trend was also observed for the Thai variety (*Z. mauritiana* var. Thai), in which fruit extracts showed the strongest antioxidant activity. Across all samples and tissues, ethanol was a more efficient extraction solvent than either water or methanol, highlighting the importance of solvent choice in the recovery of antioxidant compounds.

These findings were in agreement with previously published studies on *Z. mauritiana*. Sinan et al. [6] reported that ethanolic extracts yielded the highest antioxidant activity, which was consistent with the solvent-dependent pattern observed in the present work. Similar findings were reported by Singh et al. [35] and Kaur et al. [36], who showed that fruits generally exhibited higher antioxidant capacity than other plant tissues, an effect linked to their greater phenolic and flavonoid content. In the current study, differences among sampling locations, plant parts, and extraction procedures were evident, suggesting that antioxidant activity in *Z. mauritiana* was not governed by a single factor but rather reflects the combined influence of genetic variation, environmental conditions, and methodological choices. Similar multifactorial influences on phenolic-mediated antioxidant activity have been reported in arid-region medicinal plants [37].

### 3.4. Phenolic Compound Profile of Ziziphus mauritiana (RP-HPLC)

RP-HPLC analysis showed substantial differences in phenolic composition across different plant parts and genotypes of *Z. mauritiana* (Table 4), reflecting the chemical diversity of *Ziziphus mauritiana* Lam. as a bioactive compound source. Gallic acid was the most abundant phenolic identified, especially in locally grown material. Local *Ziziphus mauritiana* Lam. leaves contained the highest concentration at 8.77 mg/g, while the Thai variety had much lower levels. This suggested that both genetics and growing conditions strongly influenced phenolic production. Genotype–environment interactions have been known to regulate phenylpropanoid pathway activity, thereby affecting quantitative and qualitative phenolic composition [9]. Earlier work on *Z. mauritiana* has identified gallic acid as a key phenolic marker that contributed substantially to antioxidant activity [30].

Catechin, a flavan-3-ol known for its antioxidant and antimicrobial properties, occurred mainly in *Ziziphus mauritiana* Lam. fruits, reaching 6.30 mg/g in local samples. This accumulation pattern agrees with previous findings that fruits are the main storage sites for low-molecular-weight flavonoids with good bioavailability [38]. High catechin levels in fruits help explain why fruit extracts show such strong antimicrobial and antioxidant effects in earlier *Z. mauritiana* studies [39].

Caffeic acid was another significant phenolic in *Ziziphus mauritiana* Lam. extracts. Caffeic acid reached its peak in local leaves (1.93 mg/g). Though present at lower levels than gallic acid or catechin, caffeic acid is biologically significant because of its radical-scavenging and anti-inflammatory activities, which add functional value to *Ziziphus mauritiana* Lam. leaves [40]. Thai variety leaves had moderate amounts of syringic acid, suggesting that phenolic acid biosynthesis varies between genotypes. Other phenolics appeared at lower concentrations, including p-coumaric acid, ferulic acid, rutin, hesperidin, quercetin, kaempferol and apigenin. Even as minor components, these compounds matter because they can work together synergistically, boosting the overall antioxidant and antimicrobial effectiveness of complex plant extracts well beyond what individual compounds would achieve alone. Such synergistic interactions among phenolic constituents have been reported to enhance membrane disruption and oxidative stress induction in microbial cells. This kind of synergy has been well-documented in phenolic-rich materials like *Z. mauritiana* [41].

The RP-HPLC results confirmed that *Z. mauritiana* has a diverse and functionally important phenolic profile led by gallic acid, catechin, and caffeic acid. The differences between plant parts and genotypes have highlighted why choosing the right source material matters for getting maximum bioactive potential. These data supported using *Ziziphus mauritiana* Lam.—particularly fruits and leaves—as a promising natural source of antioxidants and antimicrobials for food applications [30].

### 3.5. In Vitro Antimicrobial Activity of Ziziphus mauritiana Lam. Extracts

*Ziziphus mauritiana* Lam. extracts inhibited all tested foodborne pathogens, though activity differed between plant parts and collection sites (Table 5). Baniyas leaves produced inhibition zones of 24.43 ± 0.81 mm against *E. coli* and 24.30 ± 0.70 mm against *Y. enterocolitica*, whereas bark and fruits were less effective. Bark failed to inhibit *S. aureus* in several cases. Leaves from Kuwaitat inhibited *E. coli* and *Y. enterocolitica* with zones of 23.23 ± 1.27 and 22.20 ± 1.09 mm. Pakistani accession leaves at Salamat were effective against *S. enterica* (21.83 ± 1.76 mm) and *C. jejuni* (20.17 ± 0.76 mm), performing better than bark or fruit extracts. Thai variety leaves at Abu Koriya gave the strongest inhibition of *S. aureus* (12.27 ± 2.05 mm), whereas bark and fruits showed little activity (*p* < 0.05). Overall, these findings supported leaves as the best source tissue for antimicrobial use, consistent with their higher TPC and TFC, as well as greater phenolic diversity. A positive correlation between total phenolic content and antimicrobial efficacy has been widely reported in plant-derived extracts.

Several mechanisms probably accounted for the antimicrobial effects of phenolic extracts, and these may work synergistically. Phenolics can disrupt bacterial membranes, interfere with metabolic enzymes and energy metabolism, chelate metal ions, and damage nucleic acids [42]. These mechanisms collectively impaired cellular integrity and replication processes in both Gram-positive and Gram-negative bacteria. Gallic acid, catechin and caffeic acid—major components in *Ziziphus mauritiana* Lam. extracts—have all shown these activities in previous studies.

Gallic acid has been reported to induce membrane permeability alterations in Gram-negative bacteria, while catechin interferes with bacterial enzymatic systems and energy metabolism, leading to growth inhibition [43]. Similar phenolic-mediated antimicrobial effects have been documented in meat systems treated with plant-derived extracts [44].

*Ziziphus mauritiana* Lam. leaves inhibited both Gram-negative organisms (*E. coli*, *Salmonella*, *Yersinia*) and Gram-positive bacteria (*B. cereus*, *S. aureus*), demonstrating that the phenolic mixtures have wide-ranging antimicrobial effects [45].

### 3.6. Microbiological Analysis of Chicken Meat Fortified with Ziziphus mauritiana Extracts

Microbiological assessments of chicken meat fortified with *Ziziphus mauritiana* (sidr) extracts during 15 days of chilled storage at 4 °C demonstrated a clear, concentration-dependent antimicrobial effect against spoilage and pathogenic microorganisms (Table 6).

The initial total aerobic count of fresh chicken samples was approximately 4.93 ± 0.72 log CFU/g prior to treatment. Total aerobic counts (TAC) in the control sample increased from 4.93 log CFU/g at day 0 to 8.53 log CFU/g by day 15, whereas *Ziziphus mauritiana* Lam. -treated samples showed significantly reduced bacterial growth. Samples treated with 1% *Ziziphus mauritiana* Lam. extract showed the strongest inhibition, with TAC reaching only 5.34 log CFU/g after 15 days, which indicates effective control of aerobic bacterial growth.

According to commonly accepted microbiological criteria for poultry meat, total aerobic counts exceeding 7 log CFU/g have generally been considered indicative of spoilage and sensory rejection limits in fresh meat systems [46]. These limits were frequently applied in shelf-life determination studies to define the end of acceptable microbiological quality under refrigerated storage conditions. This threshold has frequently been applied in meat microbiology to define the upper limit of acceptable sensory quality and microbial stability during refrigerated storage. In the present study, treated samples remained below this limit throughout storage, whereas control samples exceeded this threshold, confirming the shelf-life extension effect of the extract.

A similar inhibitory pattern was recorded for yeasts, molds, and enteric bacteria. The yeast and mold counts in the control samples increased steadily to 6.56 log CFU/g by day 15, while the 1% *Z. mauritiana* Lam.-treated samples limited fungal growth to 5.09 log CFU/g. Moreover, *Salmonella shigella* populations increased in the controls from undetectable levels to 7.04 log CFU/g, whereas *Z. mauritiana* Lam. fortification significantly reduced pathogen growth, with counts of 5.52 log CFU/g in the 1% treatment. Coliform counts followed a similar pattern, rising to 7.74 log CFU/g in controls but remained substantially lower in 1% *Z. mauritiana* Lam.-treated meat (5.43 log CFU/g), which confirmed improved microbiological safety during refrigerated storage. The observed microbial reduction may be associated with phenolic-mediated membrane permeability alterations and intracellular enzyme inhibition, mechanisms widely documented in foodborne pathogens [11].

From a regulatory perspective, microbiological criteria for raw poultry meat should typically require the absence of *Salmonella* spp. in 25 g samples intended for ready-to-eat use, in accordance with microbiology guidelines [47]. These regulatory frameworks have mandated the absence of *Salmonella* spp. in specified sample units as a fundamental food safety requirement. Although the present study involved artificial inoculation at high challenge levels, the significant reduction observed in treated samples demonstrated a substantial safety improvement compared with the untreated control and highlighted the potential of *Z. mauritiana* extract to contribute toward compliance with recognized microbiological safety standards. Furthermore, the maintenance of total aerobic counts below the generally accepted spoilage threshold (7 log CFU/g), as commonly reported in meat microbiology guidelines [47], reinforces the practical shelf-life extension effect of the extract under refrigerated storage conditions.

The antimicrobial activity of *Z. mauritiana* extract comes from its phenolic acids and flavonoids, which can disrupt bacterial membranes and inhibit metabolism [48,49]. Other work has shown that *Ziziphus* extracts can reduce microbial loads in meat products [50]. These findings demonstrated that 1% *Z. mauritiana* extract effectively controlled both spoilage microorganisms and pathogenic bacteria in chicken meat, supporting its application as a natural preservative for refrigerated poultry products.

### 3.7. Inhibitory Effect of Ziziphus mauritiana Extract Against Pathogenic Bacteria

**Figure 1:**
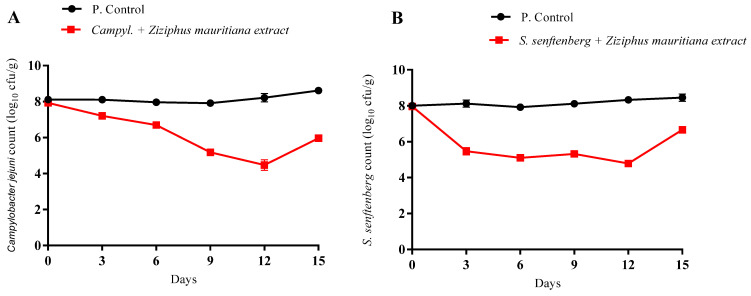
Inhibition rate of *Campylobacter jejuni* and *S. senftenberg* in chicken meat products throughout 15 days of storage at 4 °C. Inhibition rate expressed as mean values ± standard deviation. (**A**) *Campylobacter jejuni* where positive control: *Campylobacter jejuni* (P. Control); treatments: chicken meat with *Ziziphus mauritiana* extract + *Campylobacter jejuni*; (**B**) *S. senftenberg* where positive control: *S. senftenberg* (P. Control), treatments: chicken meat with *Ziziphus mauritiana* extract + *S. senftenberg*.

The inhibitory effects of *Z. mauritiana* (sidr) extract were evaluated against *Campylobacter jejuni* and *Salmonella senftenberg* in chicken meat during 15 days of storage at 4 °C (Figure 1). *Ziziphus mauritiana* Lam. extract demonstrated strong antimicrobial activity, with pathogen counts considerably lower than untreated controls.

*Campylobacter jejuni* populations in the control meat remained relatively stable throughout storage (*p* < 0.05), rising from 8.11 log CFU/g at day 0 to 8.61 log CFU/g by day 15. In contrast, *Ziziphus mauritiana* Lam.-treated samples showed progressive reductions in *C. jejuni*, declining from 7.93 log CFU/g initially to 5.96 log CFU/g by day 15. Maximum inhibition was observed on day 12, when counts decreased to 4.47 log CFU/g, reflecting substantial control of this pathogen under refrigeration. *S. senftenberg* exhibited similar behavior. Controls increased gradually from 8.00 log CFU/g at day 0 to 8.45 log CFU/g by day 15. *Ziziphus mauritiana* Lam.-treated meat had significantly lower counts, decreasing from 7.96 log CFU/g at the beginning to 5.46 log CFU/g by day 3, with minimum levels of 4.78 log CFU/g recorded at day 12. Although counts increased to 6.66 log CFU/g by day 15, the overall pattern demonstrates strong antimicrobial activity, especially during the first 12 days of storage.

The antimicrobial effects were probably due to bioactive compounds in *Z. mauritiana* extract, such as flavonoids, tannins, and polyphenols, which could damage bacterial membranes and interfere with metabolism. These compounds have been known to induce structural damage in bacterial cell envelopes and reduce ATP synthesis, ultimately suppressing pathogen proliferation under refrigerated conditions. Other researchers have reported comparable inhibition of foodborne pathogens using phenolic plant extracts [51,52]. These findings indicated that *Ziziphus mauritiana* Lam. extract has potential as a natural antimicrobial to improve microbiological safety and extend shelf life in poultry products [53].

### 3.8. Sensory Evaluation of Chicken Meat Fortified with Ziziphus mauritiana Extract

**Figure 2:**
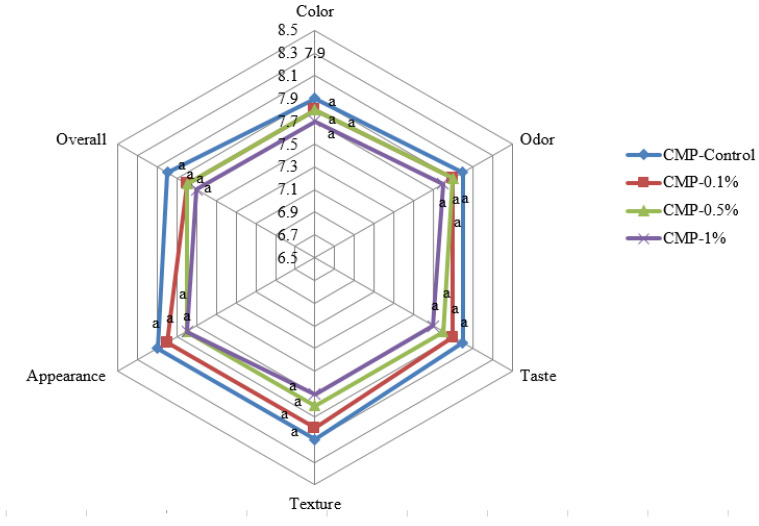
Sensory evaluation of chicken meat fortified with *Ziziphus mauritiana* extract. Negative control: chicken meat only; treatments: chicken meat with 0.1% ZML-leaves extract; chicken meat with 0.5% ZML-leaves extract; chicken meat with 1% ZML-leaves extract; where different letters indicate significant differences among treatments (*p* < 0.05), whereas identical letters indicate no significant differences. ZML: *Ziziphus mauritiana* leaf extract.

Sensory evaluation of chicken meat fortified with *Z. mauritiana* (sidr) extract showed little impact on color, odor, taste, texture, appearance, or overall acceptability (Figure 2). Sensory analysis was conducted at day 0; therefore, changes in organoleptic properties during extended storage were not evaluated. Controls scored somewhat higher for color (8.2) and texture (7.95) than *Ziziphus mauritiana* Lam.-treated samples; however, no statistically significant differences were observed among treatments (*p* > 0.05), indicating that extract incorporation did not adversely affect sensory attributes and all products received acceptable ratings. The compatibility of phenolic-rich plant extracts with meat sensory properties has been previously demonstrated when applied at optimized concentrations [25]. Even at 1% concentration, *Ziziphus mauritiana* Lam. extract did not impair sensory quality, which suggests it works well in chicken meat applications.

Sensory scores stayed fairly consistent across all *Ziziphus mauritiana* Lam. treatments, meaning the phenolics and flavonoids in *Z. mauritiana* did not create off-flavors, bad odors, or texture problems during cold storage. Drake [54] showed that adding plant extracts to meat at suitable concentrations does not harm sensory properties. The minimal sensory changes we observed support using *Ziziphus mauritiana* Lam. extract practically in foods. Comparable results have been reported by investigators working with rosemary and green tea extracts, who found that these natural compounds extended the shelf life of meat products and decreased bacterial populations without negatively affecting sensory properties [55,56]. The current study’s results indicated that *Z. mauritiana* extract functioned effectively as a natural preservative in chicken products. The extract successfully inhibited microbial growth while preserving the sensory acceptability of the treated samples. Given this combination of antimicrobial activity and maintained organoleptic quality, *Z. mauritiana* extract merits serious consideration for use in clean-label meat products, particularly where manufacturers must balance food safety requirements with consumer taste preferences. Although sensory evaluation was limited to day 0, the absence of immediate negative effects supported the feasibility of extract incorporation. Future studies should assess sensory stability throughout storage to fully establish consumer acceptability over time.

## 4. Conclusions

This study demonstrates that *Ziziphus mauritiana* extracts, particularly the ethanolic leaf extract, possessed significant antioxidant and antimicrobial activities associated with their phenolic composition. Incorporation of 1% extract into chicken meat effectively controlled spoilage and pathogenic microorganisms during refrigerated storage, maintaining microbial loads below commonly accepted spoilage thresholds while preserving initial sensory acceptability.

These findings supported the feasibility of using *Z. mauritiana* as a natural alternative to synthetic preservatives in poultry systems, aligning with clean-label and food safety objectives. Moreover, the study highlighed the potential of underutilized arid-region plant resources as sustainable sources of functional bioactive compounds. Future investigations should focus on food-grade extraction optimization, mechanistic evaluation of key phenolics, and validation under industrial processing conditions. For potential commercial implementation, further studies should address economic feasibility, toxicological safety assessment, and compliance with relevant food safety regulations and organizational guidelines to ensure industrial applicability.

## Figures and Tables

**Table 1:** Extract yield (mg/100 g) of *Ziziphus mauritiana* from different regions in Emirates.

Location	Genotype/Origin	Parts	Yield (mg/100 g)		
			Solvent for Extraction		
			Water	Ethanol	Methanol
Baniyas Research Station	Local	Leaves	8.16 ± 0.49 ^b^	8.75 ± 0.34 ^c^	9.26 ± 0.17 ^c^
		Bark	9.72 ± 0.71 ^a^	10.52 ± 0.28 ^a^	10.71 ± 0.23 ^a^
		Fruits	NA	NA	NA
Kuwaitat Research Station	Local	Leaves	7.70 ± 0.21 ^bcd^	7.93 ± 0.21 ^fg^	8.49 ± 0.17 ^de^
		Bark	8.16 ± 0.38 ^b^	8.54 ± 0.12 ^cde^	8.86 ± 0.28 ^cd^
		Fruits	6.94 ± 0.39 ^e^	7.35 ± 0.15 ^h^	7.44 ± 0.28 ^h^
	Pakistan	Leaves	8.01 ± 0.36 ^bc^	8.61 ± 0.45 ^cd^	8.96 ± 0.11 ^c^
		Bark	9.29 ± 0.24 ^a^	9.99 ± 0.28 ^b^	10.21 ± 0.30 ^b^
		Fruits	7.00 ± 0.36 ^e^	7.43 ± 0.40 ^h^	7.47 ± 0.38 ^h^
Salamat Research Station	Pakistan	Leaves	7.37 ± 0.40 ^cde^	7.67 ± 0.21 ^gh^	8.29 ± 0.17 ^ef^
		Bark	7.90 ± 0.10 ^bc^	8.25 ± 0.13 ^def^	8.52 ± 0.13 ^de^
		Fruits	6.97 ± 0.16 ^e^	7.23 ± 0.25 ^h^	7.74 ± 0.33 ^gh^
Abu Koriya, Al-Ain, Farm	Thailand	Leaves	8.18 ± 0.25 ^b^	8.68 ± 0.16 ^cd^	8.97 ± 0.16 ^c^
		Bark	7.70 ± 0.36 ^bcd^	8.10 ± 0.11 ^efg^	8.50 ± 0.30 ^de^
		Fruits	7.11 ± 0.21 ^de^	7.44 ± 0.14 ^h^	7.91 ± 0.26 ^fg^

The mean values indicated in the same columns within variables with different superscripts (a, b, c, d, e, f, g, and h) were significantly different (*p* < 0.05). Represented data are the means of triplicate ± SD. NA: Sample not available at the time of collection from the respective location.

**Table 2:** Total phenols and total flavonoids of *Ziziphus mauritiana* from different regions in Emirates.

Location	Genotype/Origin	Parts	Total Phenols Content (Mean ± SE, mg Gallic Acid, Equivalent/g DM)			Total Flavonoids Content (Mean ± SE, mg Catechol, Equivalent/g DM)		
			Solvent for Extraction			Solvent for Extraction		
			Water	Ethanol	Methanol	Water	Ethanol	Methanol
Baniyas Research Station	Local	Leaves	142.67 ± 3.05 ^c^	153.17 ± 1.75 ^bc^	155.50 ± 2.17 ^c^	3.51 ± 0.43 ^def^	4.11 ± 0.19 ^c^	4.73 ± 0.38 ^bcd^
		Bark	123.02 ± 3.00 ^d^	125.20 ± 2.30 ^d^	129.27 ± 1.61 ^d^	2.72 ± 0.20 ^fg^	3.38 ± 0.25 ^cde^	3.59 ± 0.32 ^def^
		Fruits	NA	NA	NA	NA	NA	NA
Kuwaitat Research Station	Local	Leaves	122.90 ± 3.85 ^d^	124.87 ± 2.90 ^d^	129.77 ± 2.75 ^d^	4.28 ± 0.17 ^cd^	5.22 ± 0.15 ^b^	5.57 ± 0.18 ^b^
		Bark	119.93 ± 1.67 ^d^	115.23 ± 1.36 ^e^	125.20 ± 2.02 ^d^	2.13 ± 0.32 ^g^	3.51 ± 0.25 ^cde^	4.23 ± 0.20 ^cde^
		Fruits	168.90 ± 2.15 ^a^	171.50 ± 3.96 ^a^	174.77 ± 2.59 ^a^	6.84 ± 0.67 ^a^	6.18 ± 0.50 ^a^	7.41 ± 0.16 ^a^
	Pakistan	Leaves	101.33 ± 3.05 ^e^	100.50 ± 1.32 ^f^	107.33 ± 2.08 ^e^	2.20 ± 0.09 ^g^	2.87 ± 0.22 ^ef^	4.14 ± 0.15 ^cde^
		Bark	99.43 ± 1.50 ^e^	94.36 ± 1.09 ^g^	99.23 ± 0.80 ^f^	2.13 ± 0.16 ^g^	3.05 ± 0.16 ^def^	3.46 ± 0.25 ^ef^
		Fruits	155.67 ± 3.78 ^b^	153.17 ± 1.75 ^bc^	164.23 ± 1.75 ^b^	5.20 ± 0.17 ^b^	4.08 ± 0.13 ^c^	5.88 ± 0.51 ^b^
Salamat Research Station	Pakistan	Leaves	97.66 ± 3.05 ^e^	101.67 ± 5.13 ^f^	109.83 ± 2.25 ^e^	3.63 ± 0.55 ^de^	3.02 ± 0.76 ^ef^	4.73 ± 0.98 ^bcd^
		Bark	90.66 ± 1.52 ^f^	91.16 ± 0.76 ^g^	92.70 ± 1.50 ^fh^	3.13 ± 0.25 ^ef^	2.99 ± 0.17 ^ef^	3.26 ± 0.28 ^ef^
		Fruits	151.80 ± 2.88 ^b^	156.13 ± 1.80 ^b^	159.70 ± 2.13 ^c^	4.70 ± 0.45 ^bc^	3.94 ± 0.33 ^cd^	5.16 ± 0.72 ^bc^
Abu Koriya, Al-Ain, Farm	Thailand	Leaves	88.00 ± 3.60 ^f^	93.50 ± 1.50 ^g^	96.46 ± 1.86 ^fg^	2.06 ± 0.15 ^g^	2.38 ± 0.34 ^f^	2.75 ± 0.33 ^f^
		Bark	84.43 ± 3.17 ^f^	89.93 ± 0.40 ^g^	91.39 ± 0.57 ^h^	2.17 ± 0.22 ^g^	2.78 ± 0.40 ^ef^	2.56 ± 0.48 ^f^
		Fruits	142.10 ± 1.31 ^c^	149.31 ± 0.87 ^c^	158.10 ± 1.68 ^c^	4.14 ± 0.12 ^cd^	4.03 ± 0.24 ^c^	5.13 ± 0.26 ^bc^

The mean values indicated in the same columns within variables with different superscripts (a, b, c, d, e, f, g, and h) were significantly different (*p* < 0.05). Represented data are the means of triplicate ± SD. NA: Sample not available at the time of collection from the respective location.

**Table 3:** Antioxdiant activity of *Ziziphus mauritiana* from different regions in Emirates.

Location	Genotype/Origin	Parts	Antioxidant Activity		
			Solvent for Extraction		
			Water	Ethanol	Methanol
Baniyas Research Station	Local	Leaves	58.60 ± 1.21 ^bc^	62.62 ± 1.01 ^a^	63.70 ± 1.64 ^ab^
		Bark	52.43 ± 1.60 ^d^	51.37 ± 0.76 ^b^	52.97 ± 1.32 ^cd^
		Fruits	NA	NA	NA
Kuwaitat Research Station	Local	Leaves	49.70 ± 1.08 ^d^	52.10 ± 0.90 ^b^	54.46 ± 2.39 ^c^
		Bark	43.63 ± 1.19 ^f^	45.79 ± 1.10 ^c^	48.26 ± 0.60 ^e^
		Fruits	65.50 ± 0.87 ^a^	63.85 ± 1.18 ^a^	67.55 ± 0.91 ^a^
	Pakistan	Leaves	48.54 ± 0.84 ^de^	50.63 ± 1.47 ^b^	52.36 ± 1.35 ^cd^
		Bark	48.40 ± 0.75 ^de^	49.30 ± 0.76 ^b^	49.30 ± 0.79 ^de^
		Fruits	63.96 ± 1.45 ^a^	62.76 ± 1.27 ^a^	66.51 ± 0.90 ^ab^
Salamat Research Station	Pakistan	Leaves	48.40 ± 1.13 ^de^	51.23 ± 1.59 ^b^	54.43 ± 1.14 ^c^
		Bark	44.80 ± 1.50 ^ef^	44.17 ± 3.23 ^c^	45.64 ± 1.92 ^e^
		Fruits	61.94 ± 2.77 ^ab^	62.10 ± 1.90 ^a^	65.00 ± 1.60 ^ab^
Abu Koriya, Al-Ain, Farm	Thailand	Leaves	48.93 ± 2.15 ^d^	52.87 ± 0.60 ^b^	53.91 ± 1.92 ^c^
		Bark	48.62 ± 0.87 ^de^	51.76 ± 0.77 ^b^	52.80 ± 1.81 ^cd^
		Fruits	56.70 ± 1.48 ^c^	60.47 ± 1.21 ^a^	62.77 ± 1.177 ^b^

The mean values indicated in the same columns within variables with different superscripts (a, b, c, d, e, and f) were significantly different (*p* < 0.05). Represented data are the means of triplicate ± SD. NA: Sample not available at the time of collection from the respective location.

**Table 4:** HPLC analysis of phenolic compounds expressed as amount (mg/g), present in *Ziziphus mauritiana* extract.

No	Compounds	ZML-Leaves	ZMT-Leaves	ZMP Fruits	ZMT Fruits	ZML Fruits
1.	Gallic acid	1.049	0.453	1.40	2.727	**6.430**
2.	Catechol	ND	2.065	0.382	ND	ND
3.	p-Hydroxybenzoic acid	1.788	ND	1.008	0.125	1.022
4.	Catechin	6.302	0.336	3.674	ND	ND
5.	Vanillic acid	ND	ND	0.149	0.179	0.125
6.	Caffeic acid	1.932	0.057	0.271	0.204	1.124
7.	Chlorogenic acid	4.525	0.170	0.668	22.625	ND
8.	Syringic acid	3.048	0.700	0.268	0.075	25.239
9.	p-Coumaric	0.298	0.178	0.064	0.281	0.526
10.	Ferulic	1.656	ND	0.749	ND	0.259
11.	O-Cumaric acid	0.379	0.082	0.105	0.049	0.181
12.	Rutin	28.025	2.922	2.041	0.812	1.839
13.	Hesperidin	17.504	3.52	0.220	0.485	10.13
14.	Resveratrol	ND	ND	3.453	1.805	1.798
15.	Myricetin	2.230	0.4	1.173	0.209	0.306
16.	Rosemarinic acid	2.939	0.275	0.622	0.206	ND
17.	Quercetin	8.658	0.666	2.694	0.465	ND
18.	Kaempferol	0.115	ND	0.166	0.095	ND
19.	Apigenin	0.010	0.007	ND	ND	0.007

ZML: *Ziziphus mauritiana* (local); ZMP: *Ziziphus mauritiana* Lam. (Pakistan); ZMT: *Ziziphus mauritiana* var. Thai (Thailand).

**Table 5:** Antimicrobial activity of *Ziziphus mauritiana* from different regions in Emirates.

Location	Genotype/Origin	Parts	Antimicrobial Activity					
			*E. coli*	*S. senftenberg*	*C. jejuni*	*B. cereus*	*Y. enterocolitica*	*S. aureus*
Baniyas Research Station	Local	Leaves	24.43 ± 0.81 ^a^	19.73 ± 1.54 ^b^	20.57 ± 0.40 ^a^	24.30 ± 0.70 ^a^	16.30 ± 1.06 ^a^	13.23 ± 0.87 ^a^
		Bark	15.43 ± 1.36 ^d^	11.47 ± 1.29 ^fgh^	13.17 ± 0.76 ^def^	15.30 ± 1.15 ^e^	11.30 ± 0.75 ^fg^	0.00 ± 0.00
		Fruits	NA	NA	NA	NA	NA	NA
Kuwaitat Research Station	Local	Leaves	23.23 ± 1.27 ^ab^	18.50 ± 0.62 ^b^	16.77 ± 0.75 ^bc^	22.20 ± 1.09 ^b^	13.07 ± 1.01 ^cdef^	12.03 ± 1.00 ^ab^
		Bark	15.20 ± 1.91 ^de^	12.17 ± 1.04 ^efg^	16.37 ± 1.21 ^c^	20.23 ± 1.46 ^c^	12.60 ± 0.53 ^defg^	0.00 ± 0.00
		Fruits	11.20 ± 1.31 ^fgh^	9.83 ± 1.26 ^hi^	11.73 ± 1.62 ^f^	13.33 ± 1.01 ^fg^	9.23 ± 1.37 ^h^	0.00 ± 0.00
	Pakistan	Leaves	21.03 ± 1.05 ^c^	19.17 ± 1.26 ^b^	18.27 ± 0.64 ^b^	21.50 ± 0.70 ^bc^	15.43 ± 0.60 ^ab^	11.30 ± 0.98 ^bc^
		Bark	13.23 ± 0.68 ^ef^	15.60 ± 0.53 ^c^	14.67 ± 1.33 ^d^	17.27 ± 1.25 ^d^	13.03 ± 1.05 ^cdef^	0.00 ± 0.00
		Fruits	10.83 ± 1.76 ^gh^	8.90 ± 1.15 ^i^	12.17 ± 0.76 ^f^	13.93 ± 1.01 ^ef^	11.17 ± 0.76 ^g^	0.00 ± 0.00
Salamat Research Station	Pakistan	Leaves	21.27 ± 1.62 ^bc^	21.83 ± 1.76 ^a^	20.17 ± 0.76 ^a^	17.47 ± 1.27 ^d^	14.30 ± 0.61 ^bcd^	10.17 ± 0.76 ^c^
		Bark	12.04 ± 1.00 ^fg^	14.27 ± 1.42 ^cde^	14.07 ± 0.90 ^de^	17.60 ± 0.79 ^d^	12.33 ± 1.40 ^efg^	0.00 ± 0.00
		Fruits	9.20 ± 0.75 ^hi^	8.37 ± 0.72 ^i^	12.43 ± 1.21 ^ef^	14.53 ± 0.50 ^ef^	14.47 ± 0.90 ^bc^	0.00 ± 0.00
Abu Koriya, Al-Ain, Farm	Thailand	Leaves	17.17 ± 1.04 ^d^	15.10 ± 0.95 ^cd^	14.57 ± 1.29 ^d^	12.80 ± 0.72 ^fg^	13.17 ± 0.76 ^cde^	12.27 ± 2.05 ^ab^
		Bark	10.17 ± 1.04 ^ghi^	13.23 ± 1.37 ^def^	13.03 ± 1.00 ^def^	15.23 ± 1.37 ^e^	8.93 ± 1.29 ^h^	0.00 ± 0.00
		Fruits	8.17 ± 0.76 ^i^	10.47 ± 1.36 ^ghi^	13.30 ± 0.61 ^def^	12.04 ± 1.06 ^g^	12.23 ± 1.08 ^efg^	0.00 ± 0.00

*E. coli*: *Escherichia coli* BA 12296; *S. senftenberg*: *Salmonella senftenberg* ATCC 8400; *C. jejuni*: *Campylobacter jejuni* ATCC 700819; *B. cereus*: *Bacillus cereus* ATCC 49064; *Y. enterocolitica*: *Yersinia enterocolitica* ATCC 23715; *S. aureus*: *Staphylococcus aureus* NCTC 10788. The mean values indicated in the same columns within variables with different superscripts (a, b, c, d, e, f, g, h, and i) were significantly different (*p* < 0.05). Represented data are the means of triplicate ± SD. NA: Sample not available at the time of collection from the respective location.

**Table 6:** Microbial populations of functional chicken meat products fortified with *Ziziphus mauritiana* extract during 15 days of storage at 4 °C.

Microorganisms	Chicken Meat	Time (days)					
		0	3	6	9	12	15
Total aerobic counts	Control	4.93 ± 0.72 ^aD^	5.50 ± 0.29 ^aD^	6.30 ± 0.17 ^aC^	6.47 ± 0.18 ^aC^	7.35 ± 0.11 ^aB^	8.53 ± 0.08 ^aA^
	0.1%	3.93 ± 0.17 ^bF^	4.38 ± 0.19 ^bE^	5.41 ± 0.27 ^bD^	5.86 ± 0.32 ^bC^	6.37 ± 0.16 ^bB^	7.11 ± 0.12 ^bA^
	0.5%	3.83 ± 0.08 ^bD^	3.29 ± 0.24 ^cE^	5.38 ± 0.16 ^bB^	4.18 ± 0.06 ^cC^	5.25 ± 0.14 ^cB^	6.62 ± 0.11 ^cA^
	1%	3.26 ± 0.17 ^bD^	3.03 ± 0.16 ^cD^	5.22 ± 0.11 ^bA^	3.84 ± 0.16 ^cC^	4.59 ± 0.25 ^dB^	5.34 ± 0.22 ^dA^
Yeast and mold	Control	0	0	3.42 ± 0.20 ^aD^	3.99 ± 0.11 ^aC^	5.59 ± 0.36 ^aB^	6.56 ± 0.14 ^aA^
	0.1%	0	0	2.47 ± 0.27 ^bD^	3.07 ± 0.07 ^bC^	4.15 ± 0.28 ^bB^	5.96 ± 0.13 ^bA^
	0.5%	0	0	0	2.26 ± 0.20 ^cC^	3.52 ± 0.26 ^cB^	5.51 ± 0.18 ^cA^
	1%	0	0	0	2.00 ± 0.11 ^dC^	3.21 ± 0.28 ^cB^	5.09 ± 0.21 ^dA^
*Salmonella Shigella*	Control	0	0	3.94 ± 0.39 ^aD^	4.44 ± 0.12 ^aC^	6.63 ± 0.11 ^aB^	7.04 ± 0.23 ^aA^
	0.1%	0	0	3.43 ± 0.18 ^bD^	3.96 ± 0.13 ^bC^	6.02 ± 0.13 ^bB^	6.65 ± 0.11 ^bA^
	0.5%	0	0	2.78 ± 0.12 ^cD^	3.43 ± 0.19 ^cC^	5.26 ± 0.16 ^cB^	6.24 ± 0.05 ^cA^
	1%	0	0	2.43 ± 0.13 ^cD^	2.99 ± 0.12 ^dC^	4.73 ± 0.24 ^dB^	5.52 ± 0.09 ^dA^
Coliforms	Control	0	0	5.17 ± 0.13 ^aD^	5.96 ± 0.17 ^aC^	7.35 ± 0.27 ^aB^	7.74 ± 0.27 ^aA^
	0.1%	0	0	4.32 ± 0.13 ^bD^	5.15 ± 0.13 ^bC^	6.15 ± 0.16 ^bB^	7.14 ± 0.16 ^bA^
	0.5%	0	0	3.66 ± 0.19 ^cD^	4.28 ± 0.13 ^cC^	5.26 ± 0.17 ^cB^	6.08 ± 0.09 ^cA^
	1%	0	0	3.13 ± 0.16 ^dD^	3.64 ± 0.13 ^dC^	4.43 ± 0.07 ^dB^	5.43 ± 0.18 ^dA^

Means in the same row followed by different uppercase letters are significantly different (*p* < 0.05); Means in the same column followed by different lowercase letters are significantly different (*p* < 0.05).

## Data Availability

The original contributions presented in the study are included in the article. Further inquiries can be directed to the corresponding author.
